# Utilizing supplementary high-strength suture tying over a bone bridge enhances the effectiveness of interference screw as tibial fixation method in anterior cruciate ligament reconstruction

**DOI:** 10.1186/s43019-026-00325-5

**Published:** 2026-07-14

**Authors:** Alvaro Arriaza, Pablo Miragaya, Juan Mora-Macías, Esther Reina-Romo, Pablo Blázquez-Carmona, Fernando Marco, Rafael Arriaza

**Affiliations:** 1 Instituto Médico Arriaza, A Coruña, Spain; 2 Delegación Gallega de la Mutualidad de Previsión Social de Futbolistas Españoles a Prima Fija, Santiago, Spain; 3 Department of Mining, Mechanical, Energy and Construction Engineering, Higher School of Engineering, University of Huelva, Huelva, Spain; 4 Department of Mechanical and Manufacturing Engineering, Higher School of Engineering, University of Seville, Seville, Spain; 5 Department of Mechanical Engineering and Industrial Design, Higher School of Engineering, University of Cádiz, Cádiz, Spain; 6 Departamento de Traumatología, Hospital Clínico San Carlos, Madrid, Spain; 7 Facultad de Medicina, Complutense University of Madrid, Madrid, Spain

**Keywords:** ACL, Tibial fixation, Supplementary fixation, Bone bridge, Biomechanical study

## Abstract

**Background:**

Tibial fixation is the biomechanical weak point in anterior cruciate ligament (ACL) reconstruction, with numerous techniques proposed to address this challenge. This study evaluates the utility of a simple, cost-effective system: high-strength suture tying over a bone bridge (BB). Its effectiveness as standalone fixation and as a supplement to standard interference screw (IS) fixation is compared with IS alone and supplemented with other commercial systems.

**Methods:**

A biomechanical study was conducted using six fixation methods tested on 61 specimens with bovine extensor tendons in porcine tibial models. Groups included BB alone, IS alone, and four hybrid fixation methods: IS + BB, IS + a cortical screw post (CSP), IS + a PushLock^®^ anchor, and IS + a SwiveLock^®^ anchor, with at least nine specimens per group. Specimens underwent cyclic load testing (500 cycles at 100–200 N) and pull-to-failure tests (20 mm/min) to measure displacement, ultimate load to failure, and stiffness.

**Results:**

Standalone BB fixation showed limited performance, with an ultimate load to failure of 357.81 ± 90.53 N and cyclic displacement of 14.88 ± 3.28 mm, insufficient for early rehabilitation. When combined with IS, BB significantly improved performance, achieving an ultimate load to failure of 500.55 ± 151.24 N, comparable to commercial systems, and reducing cyclic displacement to 4.82 ± 0.83 mm. Hybrid fixation methods lowered early failure rates to 10%, compared with 30–40% in single-method fixations, confirming enhanced stability.

**Conclusions:**

Hybrid fixation methods are more reliable than simple ones for tibial fixation. Combining IS with BB provides a biomechanically robust and cost-effective solution for ACL reconstruction. This simple, ecological and affordable method reduces early failure rates, minimizes cyclic displacement, and achieves outcomes comparable to commercial systems, offering a practical choice for improving ACL repair outcomes.

## Background

Anterior cruciate ligament (ACL) injuries are among the most frequent and impactful conditions in the field of sports traumatology. The global incidence is estimated at approximately 70 injuries per 100,000 person-years [1, 2], primarily clustered around professional or amateur sports. The specific incidence varies depending on the sport and level of demand, with a noted negative influence of female sex [3]. Treatment in active patients is typically surgical, involving reconstruction with a tendinous graft.

The weakest link in postoperative recovery chain, at least until complete integration, is found in the tibial fixation of the graft, which is the most frequent cause of early reconstruction failure [4]. This especially occurs in soft tissue grafts without a bone plug, such as those made from hamstring tendons, which are among the most commonly used in clinical practice. To identify the ideal tibial fixation system for ACL reconstruction grafts, both intra- and extratunnel models have been studied [5, 6]. These include methods of direct or indirect fixation, with the interference screw—a headless screw that compresses the graft against the tunnel walls—being the most commonly used. These systems are often combined to maximize their respective advantages, albeit with the risk of combining their disadvantages and introducing new challenges [7]. In fact, even a hybrid triple fixation has been proposed to minimize surgical failure rates, although without biomechanical studies to substantiate its efficacy [8].

Among the disadvantages of combining fixation systems are increased surgical time, high economic cost, and material-related discomfort. In this context, we aimed to study the resistance of a simple, cost-effective, and quick-to-implement tibial fixation system. This system is based on tying the guiding, high-strength sutures of the graft over a bone bridge carved into the anterior tibial cortex. This supplementary fixation method, used in combination with an interference screw, has been part of the routine clinical practice of the senior author (R.A.) for ACL reconstruction for the past 15 years. To date, only one biomechanical study has analyzed this technique, demonstrating significantly higher resistance compared with simple fixation methods [9].

The hypothesis of our work is that simple suture tying over a cortical bone bridge alone is biomechanically inferior to interference screw fixation and that when combined, they enhance tibial fixation in a manner comparable to hybrid fixation with other commercially available extracortical reinforcements.

## Materials and methods

### Study design

A biomechanical study was conducted to evaluate the tensile resistance of different tibial fixation systems for tendon grafts (*n* = 9–11 per group). For this purpose, a six-arm experimental design was created, incorporating six different fixation models with a minimum of nine specimens per arm. A bioequivalent setup was used, consisting of bovine digital extensor tendons grafted into porcine tibial bones to simulate human anatomical and biomechanical properties.

Ethical approval was not required for this study.

### Preparation of specimens

The specimens were obtained from a local butcher (Coren^®^). To prepare the grafts, bovine extensor tendons were carefully extracted and secured at both ends using high-strength traction sutures in a quadruple braid configuration. FiberLoop^®^ #2 (Arthrex, Naples, Florida, UUSS) sutures were used, allowing the creation of bifascicular grafts with an average diameter of 8.5 ± 0.5 mm, achieved by folding the tendon onto itself.

**Fig. 1:**
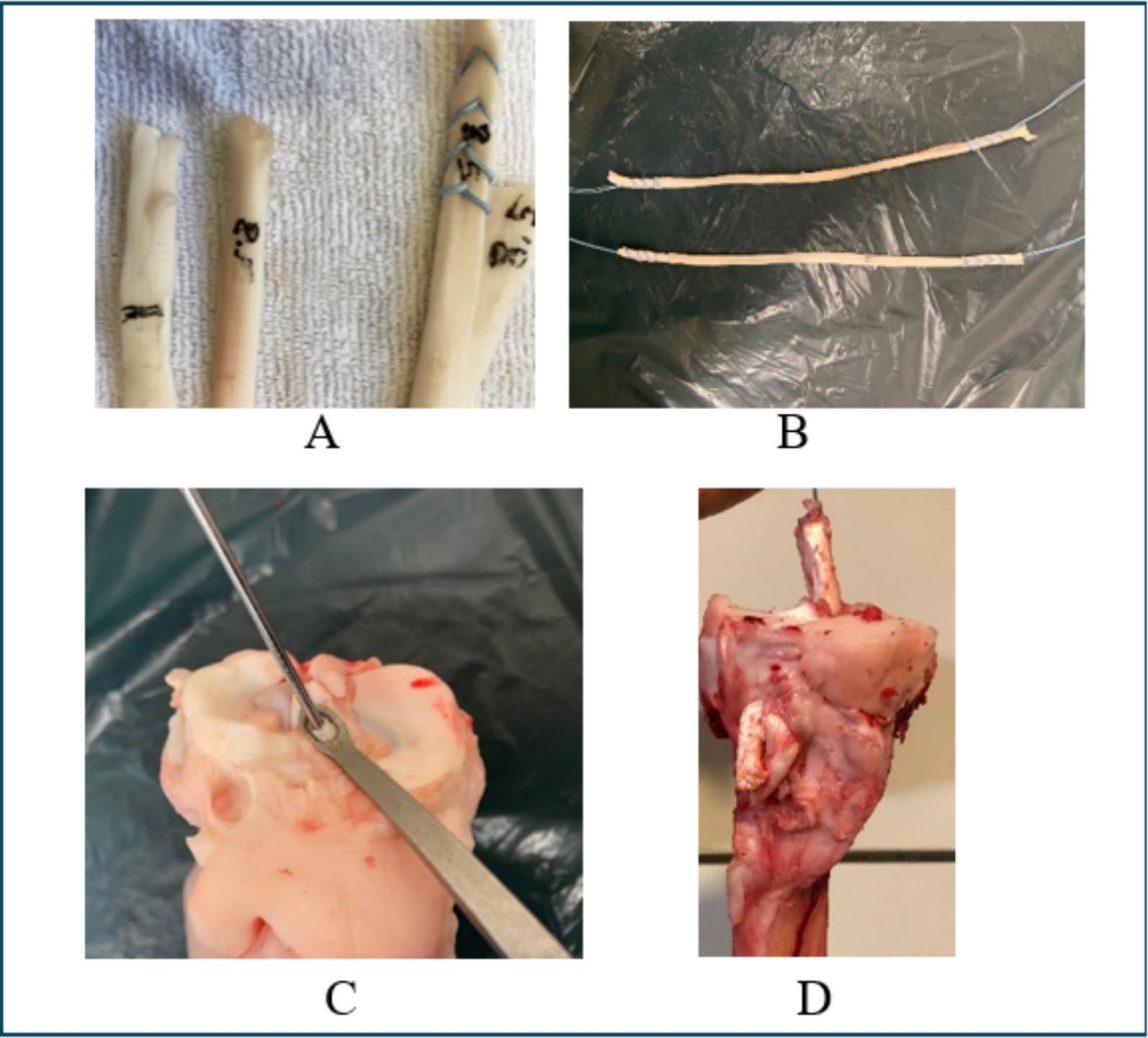
Demonstration of the graft preparation and surgical technique. Preparation of the grafts with the high-strength sutures (**A**, **B**). Anterograde drilling in the porcine tibia using the tibial guide (**C**). Example of the final preparation of one specimen (**D**)

Next, the porcine tibia was prepared to mimic the surgical procedure in humans. A tibial guide with variable angulation (Arthrex) was set at a common angle of 55°. Using this guide, an anterograde drilling was performed from the anteromedial surface of the tibia to its exit point at the anatomical insertion of the ACL on the anterior tibial spine, using a 9 mm diameter metal drill bit (Fig. 1).

**Fig. 2:**
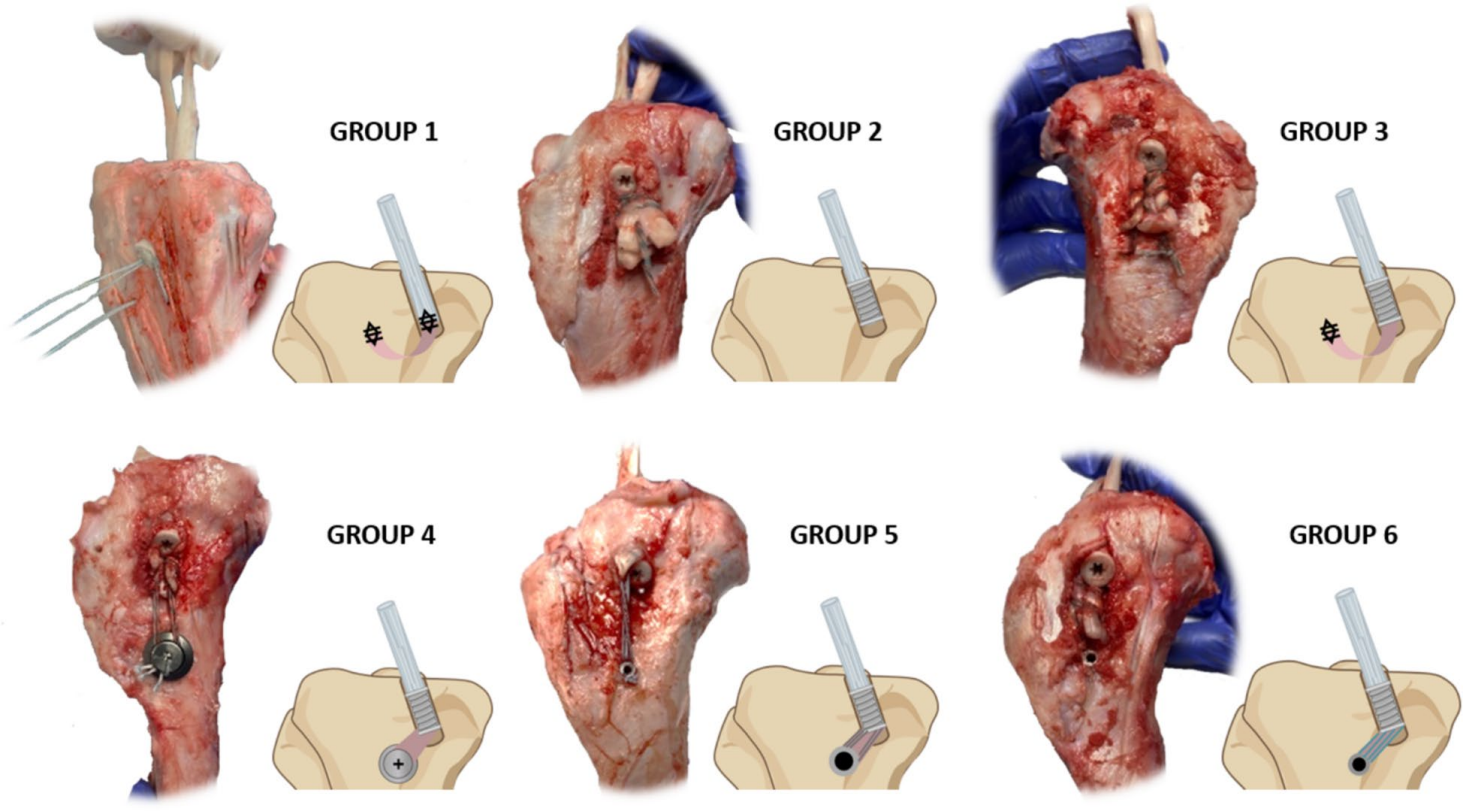
Examples of the six different methods of fixation

**Fig. 3:**
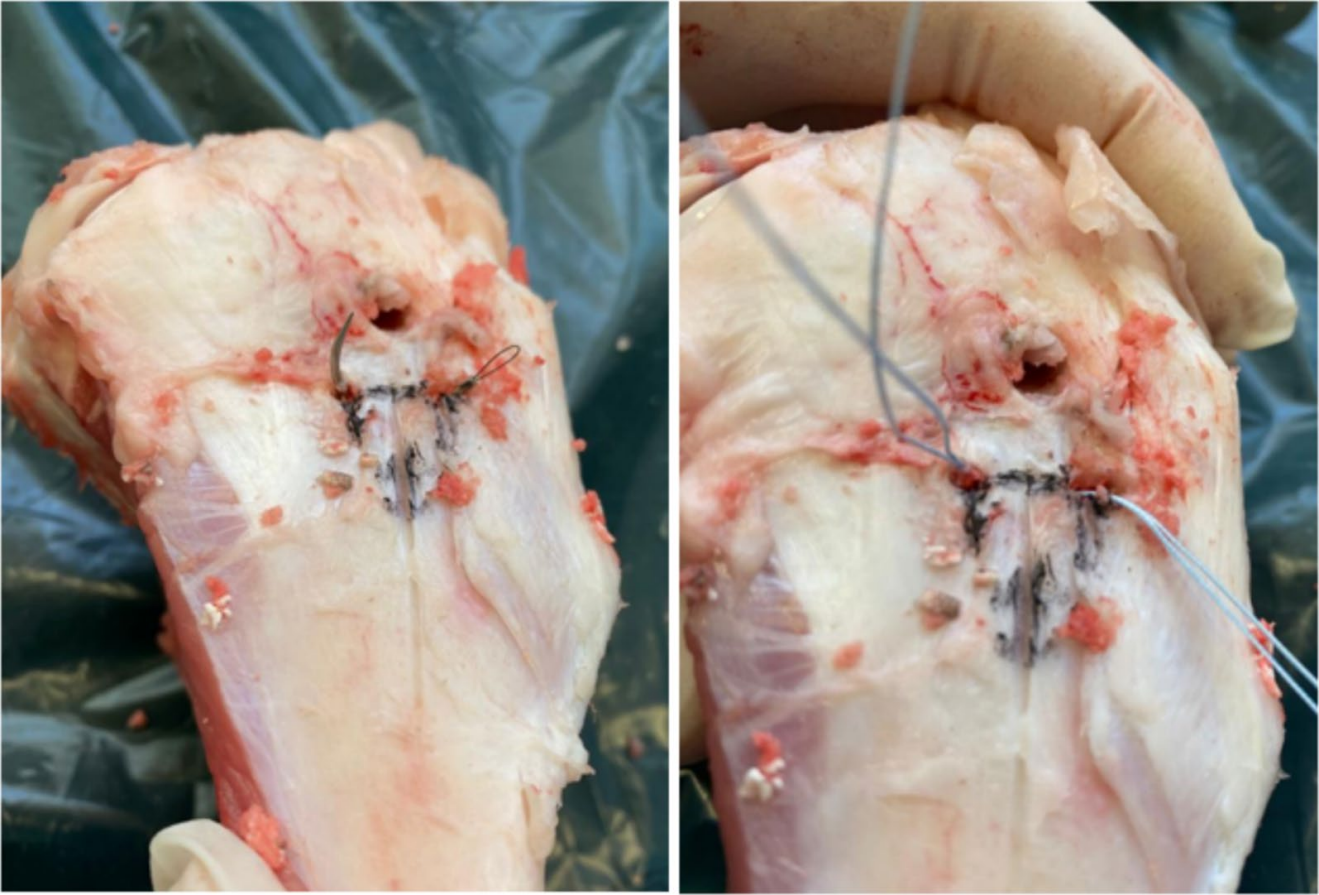
Suture passage through the bone bridge

The bifascicular grafts were introduced into the tibial tunnel via a guiding suture until their final position was achieved. Subsequently, they were randomly assigned to six groups and fixed under tension using the respective methods (Fig. 2):

- Group 1: simple suture tying over a cortical bone bridge. A double cortical perforation of the tibia was performed using a 2 mm drill bit in a converging direction, 1 cm distal to the tunnel exit, and spaced 1 cm apart horizontally. Once the graft was positioned, the two traction sutures corresponding to one tendon strand were passed through the drilled holes using a reverse needle (Arthrex) and tied with the sutures from the other strand (Fig. 3).
- Group 2: fixation with an isolated interference screw. A conical, bioabsorbable interference screw (BioComposite^®^, Arthrex, 9 × 23 mm) matching the tunnel diameter was used.
- Group 3: interference screw supplemented with suture tying over a bone bridge. Fixation combined the two methods described in groups 1 and 2.
- Group 4: interference screw supplemented with a metallic cortical screw as a post. Following initial fixation with the interference screw as described, the guiding sutures of the tendons were tied over a screw with a toothed washer used as a post, positioned distal to the tibial tunnel.
- Group 5: interference screw supplemented with guiding suture fixation using a PushLock^®^ anchor. After placing the interference screw, the high-strength sutures were tensioned and secured over a cortical bone anchor (Biocomposite PushLock^®^, Arthrex, 4.75 mm).
- Group 6: interference screw supplemented with guiding suture fixation using a SwiveLock^®^ anchor. After interference screw placement, the FiberLoop^®^ sutures were supplemented with a SwiveLock^®^ anchor (Arthrex, 5.5 mm).

For logistical reasons, the randomization and systematic assembly of all specimens were performed first, randomly dividing them into the six study arms. After the interventions and assembly, the specimens were stored in a freezer at −20 °C. Before measurements, the specimens were thawed in small groups following a protocol of 12 h at room temperature. A total of 61 specimens, divided among the six study arms, were ultimately tested, with a minimum of nine samples per arm.

### Biomechanical testing

**Fig. 4:**
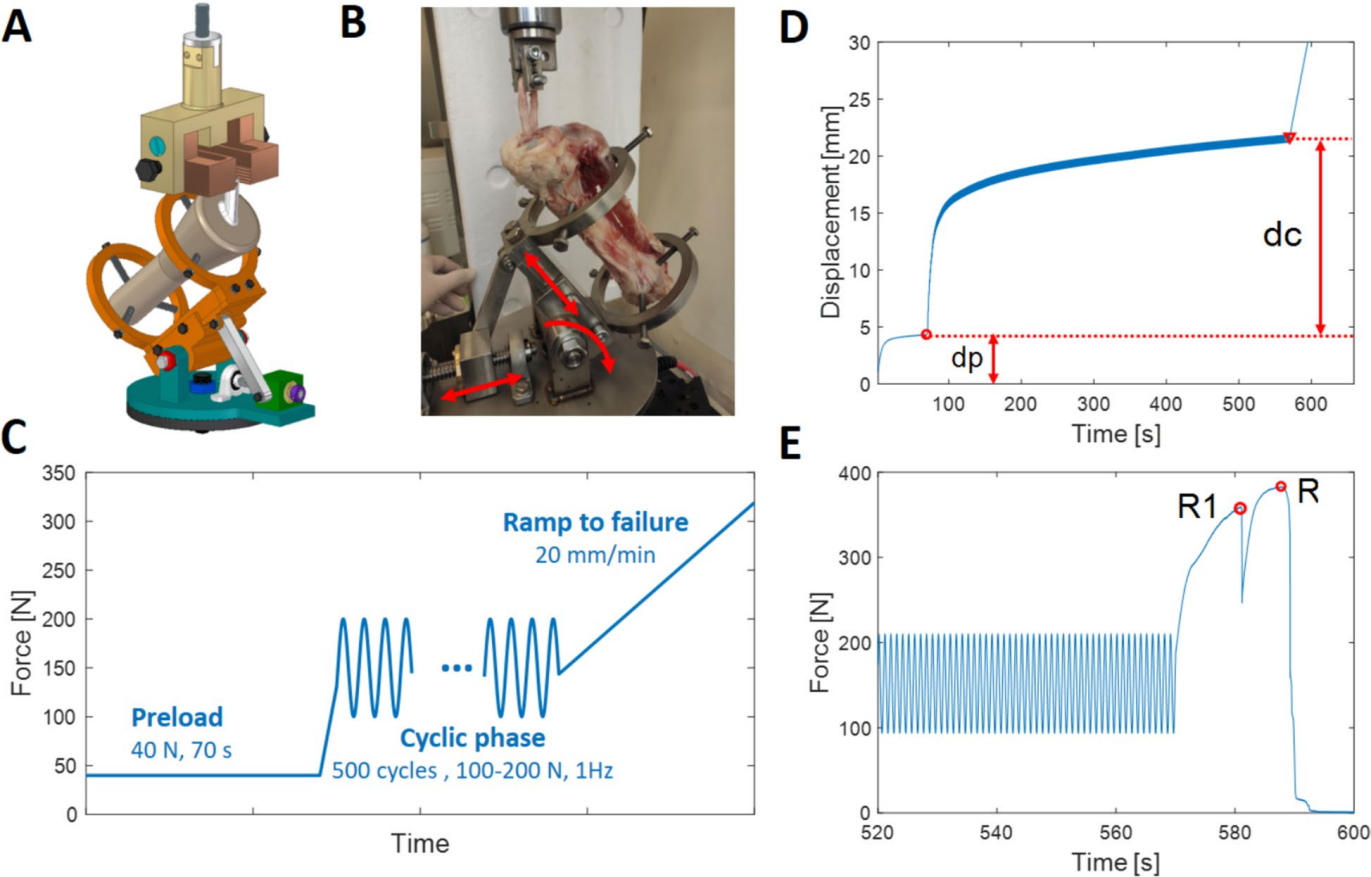
Computer model of the customized device (**A**). Biomechanical testing, indications of some of the main degree of freedom of the testing device (**B**). Displacement versus time graph during a biomechanical test (**D**). Scheme of the force applied versus time during the biomechanical test (**C**). Force versus time graph at the end of a biomechanical test (**E**)

Once thawed, the specimens were mounted on a testing machine (MTS^®^ Minibionix 858, with a load capacity of up to 15 kN, equipped with a 5 kN load cell). The bone was securely fixed using locking pins, and the looped graft was suspended using a hook-type system (Fig. 4A, B). The specimens were kept hydrated throughout the biomechanical testing by periodically spraying them with phosphate-buffered saline. Attempting to replicate the “worst possible scenario” [10], the graft and traction forces were aligned with the tibial tunnel. To ensure proper loading direction, a custom-made device was employed, allowing six degrees of freedom for specimen movement. The device comprises two circular frames with pins to fix the tibia. The block bone-frame can move along a sliding table (orange in Fig. 4A). This assembly, consisting of the bone-frame and sliding table, is capable of rotating around the horizontal axis (see Fig. 4A, B) through a manually operated worm gear mechanism. The worm gear drives a nut (highlighted in green in Fig. 4A) connected to the frame. The entire setup is mounted on a plate (shown in blue in Fig. 4A), which can rotate about the vertical axis and move within the same plane relative to the black plate, which is fixed to the load cell of the material testing machine. The device was constructed using stainless steel to ensure adequate stiffness. Calculations and validations confirmed that any displacement caused by deformation or clearance between components during testing was negligible compared with the displacements occurring at the tendon–bone fixation, which were the focus of characterization.

The biomechanical test protocol is schematized in Fig. 4C. A small preload of 5 N was initially applied to each sample, to counteract the elongation capacity of collagen—the main component of the tendon [11, 12]—and to address the potential waviness of both the tendon and the sutures when placed in the system, followed by a constant load of 40 N for 70 s. Subsequently, cyclic load testing was performed, consisting of 500 cycles between 100 and 200 N at a frequency of 1 Hz. Specimens that survived cyclic loading were subjected to a pull-to-failure test at a constant speed of 20 mm/min (Fig. 3C). Load and displacement data were recorded at 512 Hz.

As a result of the tests, several parameters corresponding to the graft-fixation-bone system were calculated: displacement during preload (DP, mm, see Fig. 4D); displacement during cyclic loading (DC, mm, see Fig. 4D); ultimate load to failure (R, N, see Fig. 4E); yield load (R1, N, see Fig. 4E), defined as the first point on the load–displacement curve where the slope clearly decreases; and fixation stiffness (K, N/mm), defined as the slope of the force–displacement graph after the cyclic loading phase. Additionally, the mode of failure for each specimen was documented.

### Statistical analysis

For the statistical analysis of the results, Matlab (MathWorks, Massachusetts, USA) and Microsoft Excel (Microsoft Corporation, Washington, USA) softwares were used. For each group, mean values and standard deviations of the variables were calculated. Differences between groups were evaluated using one-way analysis of variance (ANOVA) for independent data, with a significance level set at *p* < 0.05.

Finally, the effectiveness percentages of the different techniques were analyzed, establishing minimum criteria necessary for them to be considered satisfactory for clinical use. These criteria were determined on the basis of the literature (previous knowledge and similar published studies) and clinical experience: (a) to pass preload and cyclic loading (below 200 N) without premature failure; (b) to maintain displacement at the end of cyclic loading below 6 mm, simulating the repetitive low-load cycles typical of rehabilitation, and (c) to achieve an ultimate load to failure (R) above 300 N, estimated as the maximum force the graft might experience during the early weeks of rehabilitation.

## Results

There were no cases of iatrogenic cortical fracture during the drilling of the bone bridge. After randomization and preparation, 10 specimens were tested in group 1, 11 in group 2, 10 in group 3, 11 in group 4, 9 in group 5, and 10 in group 6. Early failures (during cyclic loading) occurred in several groups: three specimens failed prematurely in group 1, four in group 2, one in group 3, and one in group 6. Table 1 presents the mean values and standard deviations for the parameters DP, DC, R, R1, and K across the six study groups. All failures, both early and during the pull-to-failure tests, were due to suture rupture and/or graft slippage within the intratunnel fixation. None of the specimens experienced rupture of the graft itself, cortical bone bridge fracture, or dislodgement or failure of the specific fixation systems, apart from the sutures.

**Table 1:** Results of the biomechanical testing

	Group 1	Group 2	Group 3	Group 4	Group 5	Group 6
DP (mm)	3.8 ± 1.2*^23,456^	1.6 ± 0.6*^1^	1.9 ± 0.3*^14^	1.6 ± 0.4*^13^	1.9 ± 0.5*^1^	1.7 ± 0.5*^1^
DC (mm)	14.9 ± 3.3*^23,456^	4.9 ± 1.6*^1^	4.8 ± 0.8*^1^	6.9 ± 5.2*^1^	5.1 ± 1.0*^16^	4.1 ± 0.8*^15^
R1 (N)	326.4 ± 79.1	394.9 ± 105.8	371.9 ± 97.1	423.8 ± 148.5	392.0 ± 121.1	386.2 ± 127.5
R (N)	357.8 ± 90.5*^2346^	554.9 ± 129.2*^1^	500.6 ± 151.2*^1^	507.3 ± 128.6*^1^	439.5 ± 135.4	537.9 ± 110.2*^1^
K (N/mm)	152.7 ± 23.8	180.4 ± 52.0	188.9 ± 43.0	174.7 ± 27.6	182.7 ± 31.2	173.8 ± 54.9

*DP* displacement during preload, *DC* displacement during cyclic loading, *R* ultimate load to failure, *R* yield load, *K* fixation stiffness

Significant differences are indicated with an asterisk, referencing the groups specified after the asterisk

**Fig. 5:**
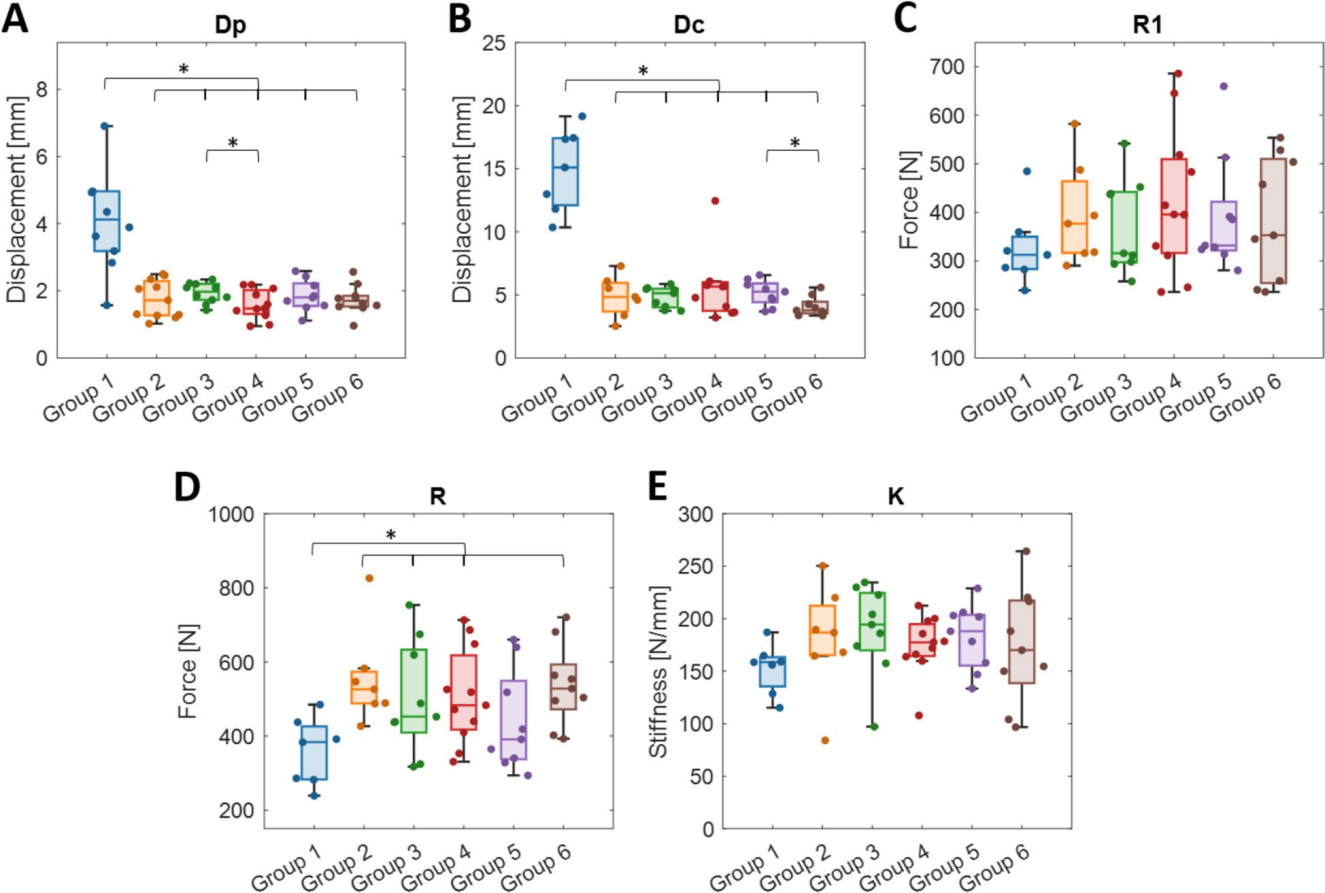
Results of the biomechanical testing. Experimental points with Box-and-whisker plots. Significant differences are indicated by a bracket connecting the corresponding groups and an asterisk. DP displacement during preload, DC displacement during cyclic loading, R ultimate load to failure, R yield load, K fixation stiffness

Results are presented in Table 1 and Fig. 5. ANOVA analysis demonstrated a significant difference in DP between group 1 (3.79 ± 1.18 mm) and all other groups. To a lesser extent, but still significantly, DP was higher in group 2 compared with group 4 (1.91 ± 0.30 mm versus 1.59 ± 0.44 mm).

The parameter DC, or displacement during cyclic loading, showed similar results. The inferior resistance to slippage in this phase of the study was evident for group 1 (14.88 ± 3.28 mm) compared with all other groups, with group 4 being the next closest (6.87 ± 5.23 mm). Despite a larger standard deviation, DC in group 4 was less than half that of group 1. Additionally, though with a higher *p* value, group 5 had a significantly higher DC than group 6 (5.13 ± 1.00 mm versus 4.07 ± 0.77 mm).

Regarding R, or ultimate load to failure, group 1 (357.81 ± 90.53 N) once again showed significantly lower values compared with all other groups except group 5 (439.45 ± 135.35 N). For yield load (R1) and stiffness (K), no statistically significant differences were observed between groups.

**Fig. 6:**
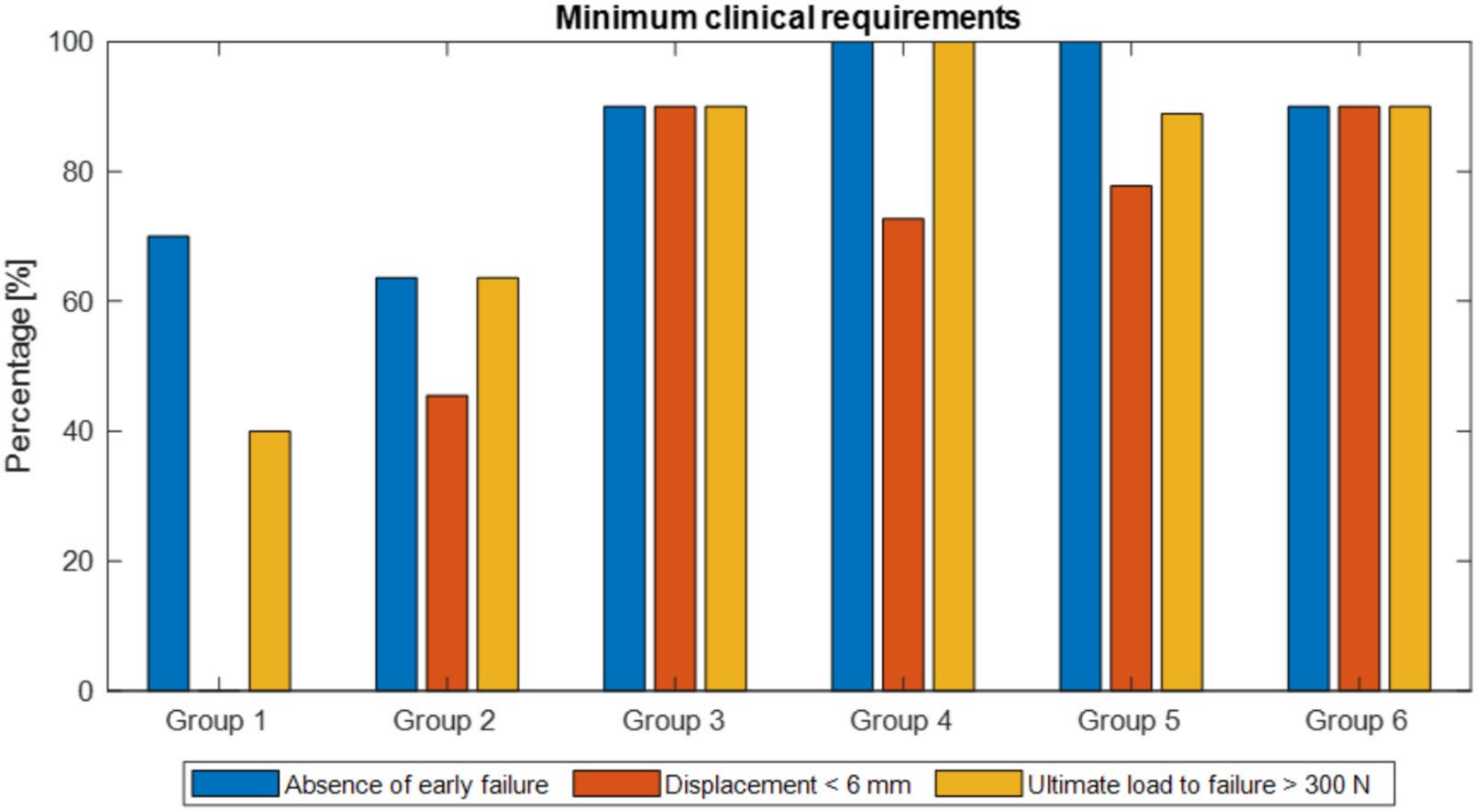
Effectiveness percentages for meeting the minimum clinical requirements

Finally, in terms of the effectiveness percentages for meeting the minimum clinical requirements (absence of early failure, displacement under 6 mm at the end of cyclic loading, and ultimate load to failure exceeding 300 N), several observations stood out (Fig. 6): (a) only specimens in groups 4 and 5 surpassed the cyclic loading phase without early failures in 100% of cases, with groups 3 and 6 closely following at 90%. The effectiveness of standalone fixation systems was markedly lower; (b) graft displacement exceeded 6 mm in all cases in group 1. The best results were found in groups 3 and 6, where only 10% of specimens experienced excessive displacement; (c) only group 4 achieved an ultimate load to failure > 300 N in all cases. Groups 3, 5, and 6 had approximately 90% success in meeting this criterion and (d) there was a substantial overall difference in meeting minimum requirements between hybrid fixation groups and standalone fixation systems.

## Discussion

The primary finding of this study is that hybrid fixation systems for ACL reconstruction with tendon grafts are biomechanically stronger, demonstrating greater reliability against premature failure and graft slippage, as well as higher resistance to ultimate load failure, regardless of the method used to supplement fixation.

A six-arm biomechanical study was performed, consistent with the design of similar comparative studies [5, 6], utilizing porcine knees and bovine extensor tendons, which are comparable and bioequivalent to human tissues, making them suitable for biomechanical studies applicable to the human knee [14–18].

In 1984, Noyes et al. [19] estimated that the maximum load supported by an ACL during daily activities could reach up to 450 N. On the basis of this data and the 300 N maximum estimated during rehabilitation exercises in the first postoperative weeks, the results of our study (Table 1; Fig. 5) indicate that the resistance capacity of an isolated system based on suture tying over a bone bridge (group 1), as proposed here, might be insufficient to meet the demands of an accelerated rehabilitation protocol, even with carefully selection of exercises [20].

Other studies have described additional complications associated with cortical fixation relying solely on high-strength sutures, such as the so-called “windshield wiper effect” [21, 22]. In our study, with 30% early failures (Fig. 6), a cyclic displacement (dc) of 14.88 mm, and an ultimate failure load of 357.81 N (Table 1), the results for a single fixation system with this arrangement are unacceptable in our setting.

The simplicity of placement and the encouraging results reported in many studies with interference screw fixation [23] have established it as the standard system for many orthopedic surgeons. However, the high rate of early failures observed in our study in the group 2 (Fig. 6) was striking. If this proportion were translatable to clinical practice, up to 36% of grafts surgically fixed in this manner could be at risk of early postoperative failure if subjected to relatively high loads, such as those used in current accelerated rehabilitation protocols [24].

A plausible hypothesis to explain this finding could be that the tibial tunnel may have slightly dilated during the freezing process. In our study, for logistical reasons, assembly was performed first, followed by freezing. Although a single freeze–thaw cycle at −20 °C is not thought to alter the biomechanical properties of bone and tendons [26–30]—and this method is the most commonly used in biomechanical studies owing to the difficulty of working with fresh specimens—the effect of freezing a bone tunnel containing an interference screw has not been studied. It remains unknown whether this could impact the tunnel, even if the mechanical properties of the bone itself are unaltered. In this regard, we found only one study in which the freezing sequence matched ours, published by Vaquero-Martín et al. [31]. In their results, the authors themselves reflect on the lower fixation strength observed in their study compared with other comparable works, where the freezing–thawing and assembly process followed the more conventional protocol (approximately 200 N versus 500 N).

If this were the case, early failures should be similarly distributed across groups 2 through 6. The fact that this did not occur highlights the safety provided by combining intratunnel fixation with cortical supplementation for load distribution. This combination appears beneficial in clinical scenarios, such as osteoporotic bone [32], drill wobble, or even oblique insertion of the interference screw, among other reasons. On the other hand, early failures due to graft slippage within the tunnel may have been related to greater mismatches between graft and tunnel diameters. Although the average graft diameter was 8.5 ± 0.5 mm, slight variations were present. The smallest diameter found was 7.5 mm and the largest was 9 mm. Most of the tendons had a diameter (once folded) of 8 or 8.5 mm.

In groups 3, 4, 5, and 6, the rate of failures during cyclic loading was drastically reduced to one or no cases per group (0–10%), even despite the potential tunnel dilation effect. Moreover, these groups showed minimal differences in displacement and resistance. The results are much more encouraging and acceptable for surgeons, reinforcing the initial theory of the study in favor of cortical supplementation.

Several studies have been published on hybrid tibial fixation. The systematic review by Balazs et al. [7] concluded that hybrid tibial fixation provided better initial graft strength and less knee laxity compared with single-method fixation. However, no significant differences in clinical outcomes were observed after 1–3 years of follow-up between patients undergoing hybrid tibial fixation and those receiving single-method fixation. This raises the question of whether the added cost of employing a second implant for fixation supplementation is justified.

The various supplementation methods that were evaluated in our study, all commonly used in human clinical practice, each have their own advantages and disadvantages. The screw with a washer (group 4), for example, is a relatively inexpensive system, quick and easy to place, but associated with a notable rate of complications related to material discomfort [33]. Anchor systems, such as the PushLock^®^ (group 5) and SwiveLock^®^ (group 6), have also been studied for this purpose [35–36] and, while effective and widely used for other indications with fewer material-related complications, are somewhat more complex to use and add significant cost to the procedure.

By the other hand, ACL reconstruction surgery alone produces an estimated 47 kg of CO_2_ equivalents [37]. In this light, avoiding the use of new plastic or metal implants and making full use of materials already present in the original procedure adds significant ecological value to the technique—beyond its economic implications.

The authors believe it is of utmost interest, and crucial to our study, to analyze the results concerning the effectiveness percentages of the various setups in meeting the minimum criteria we considered necessary to classify a system as safe (Fig. 6).

According to the International Knee Documentation Committee (IKDC), anterior laxity measured using a Lachman test greater than 5 mm compared with the contralateral knee is considered pathological [38, 39]. Assuming that the anterior angle formed by the tibial plateau and the diaphyseal cortex is approximately 90°, the drilling angle is 55°, and the tibial tunnel entry point on the plateau is fixed, and estimating the tibial tunnel length at around 40 mm, we can calculate, using the Pythagorean theorem, the impact on the increase in the length of the opposite side (i.e., sagittal translation or anterior laxity) resulting from displacement in the tibial tunnel over the original tunnel. A graft slippage of 6 mm from its tibial fixation would correspond to an increase of 3.44 mm in the opposite side, which translates to an equivalent increase in anterior laxity. When slippage exceeds 7 mm, the expected values for anterior laxity increase beyond 4 mm, approaching the pathological thresholds established by IKDC criteria. On the basis of these trigonometric calculations and the existing literature, we decided to adopt 6 mm of maximum slippage as one of the minimum safety criteria for our study.

Although including a minimum criterion on the basis of fixation stiffness was initially considered, no solid data in the literature were found to establish reliable recommendations. Some studies advocate for the stiffest possible fixations [41–42], while others suggest that lower stiffness is healthier for the joint [43, 44], and some even argue that systematic inaccuracies in usual measurement methods make stiffness data unreliable [45]. This controversy ultimately dissuaded us from using stiffness as a minimum criterion.

In this study, groups 1 and 2 fell far short of the required standards. By contrast, the groups incorporating supplementary fixation (groups 3–6) demonstrated more consistent and reliable results. While groups 4 and 5 successfully avoided any early failures, groups 3 and 6 showed very similar and homogeneous outcomes, with a success rate of no less than 90% across all evaluated parameters.

Although at the beginning of this study we found only one article published in 2008 presenting this concept as a technical note [46], since 2022 several publications have emerged supporting the idea of tying sutures over a cortical bone bridge to supplement fixation. Some of these studies propose vertical holes [9], others transverse ones [47], and one uses patellar tendon grafts in bone–tendon–bone configuration [48]. However, all of them reach the same conclusion regarding the simplicity, effectiveness, and lack of complications associated with this technique.

The only biomechanical study comparable to ours is that of Peez et al. [9]. Remarkably, some magnitudes obtained in their study significantly differ from and surpass those in ours—for example, they demonstrated a maximum resistance of 1166.9 ± 99.1 N for the hybrid construct of an interference screw and bone bridge. Some explanations include the possible widening of the tibial tunnel in our setting, the application of traction forces in their study, that are not exactly oriented with the worst possible scenario, and their use of porcine flexor digitorum tendons, which were precisely adjusted to the 9 mm tunnel diameter in a monofascicular configuration similar to a quadriceps tendon graft, while in our study, bovine extensor digitorum tendons were used in a setup similar to a pes anserinus graft, with variable diameters averaging 8.5 mm and no adjustment of the tunnel diameter to the exact graft diameter, instead using a 9 mm tunnel. This approach more closely resembles clinical practice in humans. Nevertheless, their biomechanical analysis aligns with ours in concluding that hybrid systems are significantly superior to single systems, with no substantial differences between supplementation with or without material (button versus bone bridge) in terms of ultimate resistance or elongation/slippage.

### Limitations of the study

This work is a biomechanical study, not a clinical one. Randomized clinical trials would be necessary to reliably translate the information obtained into real-world patient outcomes.

As mentioned previously, the freezing and assembly protocol used in this study was not the standard approach, and its application in this manner has not been studied. Therefore, the possibility of a dilation effect in the predrilled tunnels cannot be excluded.

Finally, the tibial tunnel was uniformly drilled to 9 mm in all cases. While the graft diameters were reasonably consistent, averaging around 8.5 mm, slight differences were present that were not recorded. These variations could potentially explain a greater propensity for slippage in some specimens.

## Conclusions

Hybrid fixation methods are more reliable than simple ones for tibial fixation. Combining an interference screw with a bone bridge provides a biomechanically robust and cost-effective solution for ACL reconstruction. This simple, ecological, and affordable method reduces early failure rates, minimizes cyclic displacement, and achieves outcomes comparable to commercial systems, offering a practical choice for improving ACL repair outcomes.

## Data Availability

The data that support the findings of this study are partially included in Table 1, and are available from the corresponding author upon request.
